# Comprehensive plasma steroidomics reveals subtle alterations of systemic steroid profile in patients at different stages of prostate cancer disease

**DOI:** 10.1038/s41598-024-51859-1

**Published:** 2024-01-18

**Authors:** Sergey Girel, Pavel A. Markin, Elena Tobolkina, Julien Boccard, Natalia E. Moskaleva, Serge Rudaz, Svetlana A. Appolonova

**Affiliations:** 1 School of Pharmaceutical Sciences, University of Geneva, 1211 Geneva 4, Switzerland; 2 World-Class Research Center Digital Biodesign and Personalized Healthcare, I.M. Sechenov First Moscow State Medical University, 119435 Moscow, Russia; 3 Laboratory of Pharmacokinetics and Metabolomic Analysis, Institute of Translational Medicine and Biotechnology, I.M. Sechenov First Moscow Medical University, Moscow, Russia; 4 I.M. Sechenov First Moscow State Medical University, 119435 Moscow, Russia

**Keywords:** Prostate cancer, Prognostic markers, Bioanalytical chemistry, Mass spectrometry

## Abstract

The steroid submetabolome, or steroidome, is of particular interest in prostate cancer (PCa) as the dependence of PCa growth on androgens is well known and has been routinely exploited in treatment for decades. Nevertheless, the community is still far from a comprehensive understanding of steroid involvement in PCa both at the tissue and at systemic level. In this study we used liquid chromatography/high resolution mass spectrometry (LC/HRMS) backed by a dynamic retention time database DynaSTI to obtain a readout on circulating steroids in a cohort reflecting a progression of the PCa. Hence, 60 relevant compounds were annotated in the resulting LC/HRMS data, including 22 unknown steroid isomers therein. Principal component analysis revealed only subtle alterations of the systemic steroidome in the study groups. Next, a supervised approach allowed for a differentiation between the healthy state and any of the stages of the disease. Subsequent clustering of steroid metabolites revealed two groups responsible for this outcome: one consisted primarily of the androgens, whereas another contained corticosterone and its metabolites. The androgen data supported the currently established involvement of a hypothalamic-pituitary–gonadal axis in the development of PCa, whereas biological role of corticosterone remained elusive. On top of that, current results suggested a need for improvement in the dynamic range of the analytical methods to better understand the role of low abundant steroids, as the analysis revealed an involvement of estrogen metabolites. In particular, 2-hydroxyestradiol-3-methylether, one of the compounds present in the disease phenotype, was annotated and reported for the first time in men.

## Introduction

Prostate cancer (PCa) is the second most frequent malignancy in men after lung cancer. It is a leading cause of male mortality in Africa, the Caribbean and South America and the second reason for male deaths in the United States^1,2^. Early diagnostic readout concerning prostate gland diseases is greatly desired to anticipate or prepare clinical intervention thus reducing the associated costs and improving patients’ quality of life. On top of that, prognostic approach could improve discovery of early-stage cases of precluding prostate gland diseases, such as benign prostate hyperplasia (BPH) and prostate intraepithelial neoplasia (PIN)^3^. The former is described as a nonmalignant enlargement of the prostate gland in ageing men caused by hyperplastic growth of fibromuscular tissues of the transition zone and periurethral area^4,5^. Although causal link between BPH and PCa is not clear to the date, there is a strong association between both conditions. Epidemiological data suggest a positive effect of BPH on a risk of developing PCa. Hence proposed models include influence of chronic inflammation, metabolic and hormonal factors^6,7^. In turn, PIN is the abnormal premalignant proliferation within preexisting ducts featuring cytologic changes mimicking cancer. PIN also possesses a high predictive value for a development of subsequent PCa, especially if jointly considered with age and prostate serum antigen levels^8^. Similarly to BPH, PIN is sensitive to the changes in hormonal environment. For example, marked decrease in the prevalence and extent of PIN were observed in men, undergoing androgen deprivation therapy^9,10^. Finally, histologically identifiable cancer lesions are presumably formed either from PIN loci or via proliferative inflammatory atrophy. In this case, an inflammatory cell mediated oxidation stress is suggested to be a key carcinogenesis driver^11^.

Metabolomics has been demonstrated as a powerful tool to discover and follow disease dynamics since the time of its inception and widespread development. The backbone of metabolomics is the comprehensive analysis of endogenous small molecules, or metabolites. The latter has been already proven to be informative for multiple pathophysiological conditions, including various types of cancer. As example, changes in the levels of tricarboxylic acids, amino acids, fatty acids, polyamines and steroids were also reported for PCa. The interested reader can consult the following articles^12,13^.

**Figure 1:**
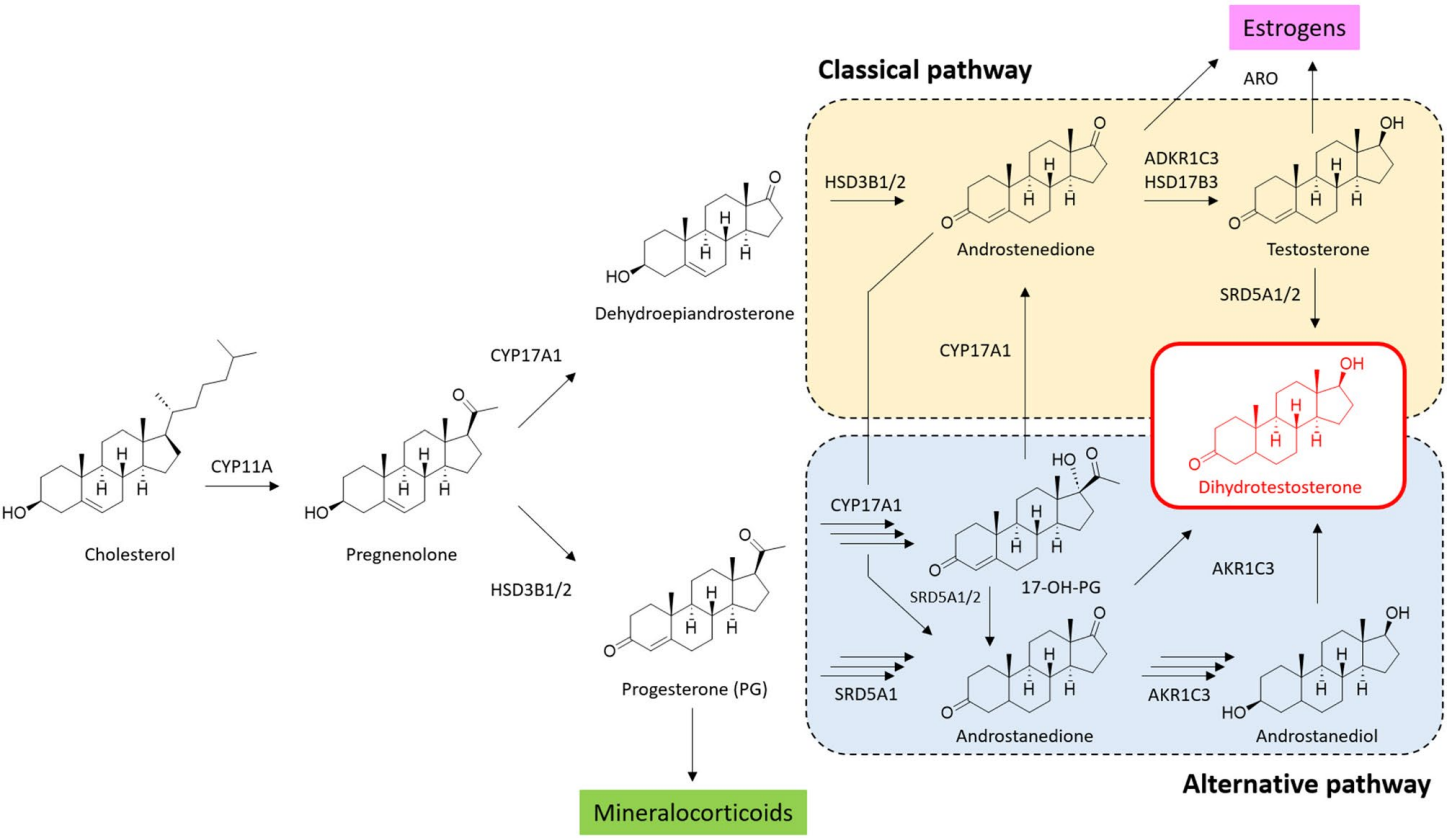
Classical and backdoor pathways of dihydrotestosterone biosynthesis in course of steroidogenesis (adapted from Ref.^19^).

In humans steroids are typically synthesized in the adrenals and gonads, although some neuroactive steroids can also be produced by brain tissues^14^. Steroids play an essential role in the differentiation and functional regulation of target cells via specific effects caused by genomic and non-genomic mechanisms. The former is mediated by hormone-specific nuclear receptors, whereas the latter are characterized by rapid effects of short duration believed to be mediated by a cell membrane^15^. High levels of steroid hormones, as well as their precursor cholesterol, are considered to be a risk factor for the development of urological diseases^16^. In addition, several studies have reported increased levels of sex hormones and steroid precursors in patients with PCa^17^. Specific families of steroid hormones, such as androgens, are of a special interest in case of PCa, as they play key roles in an oncogenic process. Testosterone (T) produced by Leydig cells in testes can directly activate androgen receptor (AR). This leads to complex proliferative and angiogenic events. On top of that, activation of the AR upregulates antiapoptotic factors, further contributing to cancer cell survival. Moreover, T in prostate tissue can be converted to a more potent metabolite dihydrotestosterone (DHT). It has a better affinity to the AR, thus increasing the rate of cancer development^15,18^. To summarize, extended profiling of steroids at both systemic and tissue levels is of great interest for early disease discovery and better understanding of underlying molecular mechanisms. A brief outline of steroidogenesis with a specific focus on DHT biosynthesis is presented in Fig. 1.

A plethora of analytical methods were developed to investigate the steroid profile in humans. These include, but not limited to, immunoassays (IA), gas (GC) and liquid chromatography (LC), usually coupled to mass spectrometry (MS). Historically, IA were considered as a method of choice in steroid analysis due to favorable costs and simple operation. However, antibodies exhibit intrinsically low specificity to small molecules, leading to a crosstalk with different matrix constituents and lower analytical accuracy. On top of that, issues with interoperability between labs and sensitivity towards important species such as testosterone and estradiol were reported. Therefore, quantitative analysis of steroids in biological matrices is typically achieved by triple quadrupole (QqQ) instruments due to their superior sensitivity and high specificity. Among the downsides of MS-based approaches are potentially high equipment and operation costs, along with a necessity of development of complex calibration approraches^20,21^.

The data obtained from QqQ include only a predefined set of compounds and is therefore not suitable for a generation of biological hypotheses. On the other side, modern high-resolution MS, such as time-of-flight (ToF) and Orbitrap systems, are capable to provide information on a broad set of compounds in biological sample simultaneously both in quantitative (with a suitable calibration approach) and qualitative fashion^22^ with albeit higher, but still sufficient levels of detection. On top of that, fast and flexible fragmentation options provided by HRMS instrumentation enable putative assignment of chemical identities of unknown compounds and downstream metabolites (ex. phase II transformations). Overall, LC/HRMS analysis remains a preferred approach in the field of biomarker discovery. Steroids, however, possess a variety of subtle structural isomers based on gonane backbone, for example, differing only in the position of one -OH group. It is thus hardly possible to differentiate these compounds based on accurate mass (AM) and/or fragmentation pattern only. An orthogonal information needed for univocal structural assignment can be provided either by altering the fragmentation mode or by the retention time (RT) dimension via LC.

**Figure 2:**
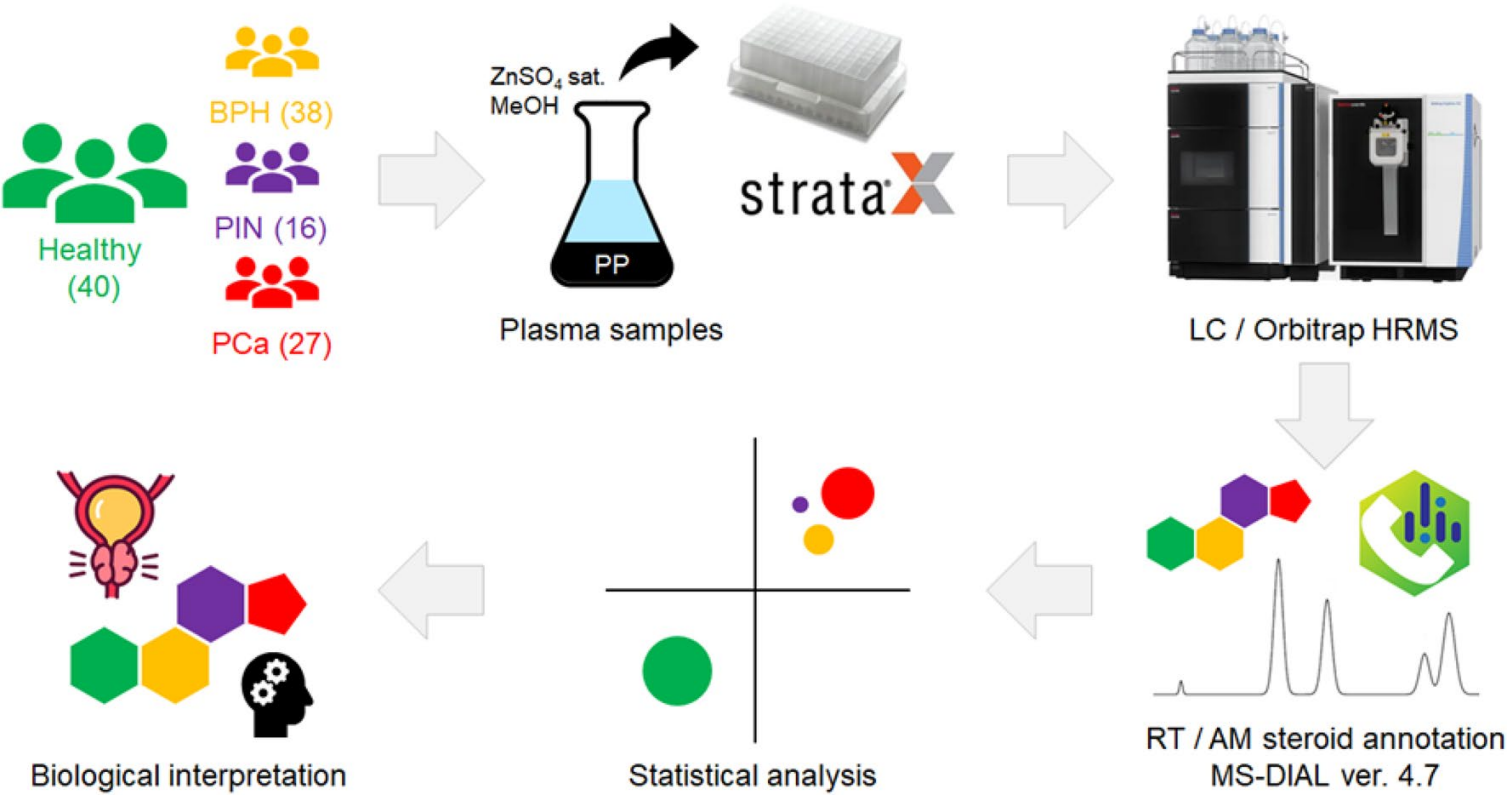
Untargeted LC/HRMS-based steroidomics pipeline to assess alterations of the extended steroid profile in patients with different stages of prostate cancer. Figure uses an image from Backwoods (flaticon.com).

In the present work, semi-targeted measurements of the plasma metabolome of patients exhibiting different stages of PCa development *vs* their healthy counterparts were performed to obtain a comprehensive snapshot of a steroidome. Orbitrap HRMS technology and the steroid compound database DynaSTi based on structure-retention time relationships (SRR) model were employed therein to annotate steroid compounds based on their RT/AM values. Thus, derived data provided an extended steroid profile, which was evaluated for an applicability to assess the status of PCa progression (Fig. 2).

## Materials and methods

### Ethical considerations

The Ethics Committee at I.M. Sechenov First Moscow State Medical University in Moscow, Russia, approved this research (Document #05-17, April 2017). Before participation in the study, each volunteer provided written and signed informed consent. The study adhered to the ethical principles outlined in the Declaration of Helsinki for conducting medical research involving human subjects^23^.

### Materials

Methanol and acetonitrile “LiChrosolv Hypergrade for LC–MS” were purchased from Merck (Darmstadt, DE). Microelution Strata-X PRO 96-well plates were received from Phenomenex (Torrance, USA). Formic acid Optima LC–MS grade and LC–MS grade water were obtained from Fisher Chemicals (Zürich, CH). Zinc sulfate heptahydrate and phosphoric acid were supplied by Sigma-Aldrich (Buchs, CH).

### Sample collection

Patients were recruited at the Research Institute of Urology and Reproductive Health (Sechenov University). Individuals were classified based on biopsies. The patients included in the study were age-matched: 67 (53–80) for PCa; 64 (52–80) for BPH; 66 (54–75) for PIN; and 64 (55–77) for controls; data presented as median (min–max). Prostate hyperplasia group was defined by an absence of caverns, poorly differentiated cells, or areas with bad differentiation; prostatic intraepithelial neoplasia group demonstrated some areas of poorly differentiated cells with several caverns; PC group biopsies contained multiple areas of badly differentiated cells with multiple caverns. Blood plasma samples were taken from a total of 27 patients with PCa, 38 patients with BPH, and 16 patients with PIN accompanied by 40 healthy controls. Venous blood samples of 5 mL were obtained in the morning, following a minimum of 8 h of fasting, using heparinized tubes. After collection, the specimens were immediately centrifuged at 5000 rpm for 10 min at 4 °C to obtain plasma. The plasma samples were kept frozen at 80 °C.

### Sample preparation

For protein precipitation plasma (100 μL) was added to 200 μL of MeOH:H_2_O (4:1 v/v) with 89 g/L ZnSO_4_ analogically to^24^. After mixing the samples were centrifuged at 14900 g (10 min, 4 °C). Supernatant (200 μL) was diluted with 200 μL of 4% phosphoric acid. This sample solution was quantitatively loaded onto a microelution Strata-X PRO 96-well plate (Phenomenex, Torrance, USA) using a negative pressure manifold. The loaded samples were consecutively washed with 100 μL of water and 100 μL of 10% MeOH. Elution was performed twice with 100 μL of 95% MeOH followed by two steps with 100 μL of MeOH/MeCN (1:1, v/v). The combined eluents were then evaporated to dryness under low pressure using miVac Quattro concentrator (Genevac, USA). Thus prepared samples were kept frozen at 80 °C. Reconstitution was achieved with 100 μL of 50% MeCN and 5 μL of the resulting solution were injected onto a chromatographic column for the analysis.

### Analytical setup

Chromatographic separation was performed on a Kinetex C_18_ column (2.1 × 150 mm, 1.7 μm; Phenomenex, Torrance, USA) in a Vanquish Horizon UHPLC system (Thermo Scientific, Waltham, USA) composed of Vanquish binary pump H, HT split sampler and heated column compartment. The system delay volume was 35 μL. Autosampler and column compartment temperatures were set to 5 and 30 °C, respectively. The separation was adapted from Ref.^25^ via adjustment of the gradient table. Briefly, the mobile phases were H_2_O + 0.1% FA (A) and MeCN + 0.1% FA (B). The gradient ran from 2 to 100% B in 14 min, with a washing phase of 3 min and subsequent equilibration phase of 8 min. The column compartment was kept at 30 °C in a forced air mode. The detection was carried out using an online hyphenated Orbitrap Exploris 120 (Thermo Scientific, Waltham, USA) mass spectrometer equipped with a heated electrospray OptaMax NG probe. The analysis was performed using the positive polarity at a scan range of 180–600 Da with resolution of 120,000@m/z200 (profile data, IT 180 ms, AGC 100%) and default ion source settings for a flow rate of 300 μL/min. Internal mass calibration was performed in scan-to-scan mode by an embedded EasyIC module using fluoranthene radical ions. External calibration was performed prior to measurements using a Pierce calibration mixture (Thermo Scientific, Waltham, USA) according to manufacturer specifications.

### Data processing

Centroiding of mass spectra (.RAW) was performed using a native algorithm (Thermo RawFileReader). The output (.MZML) was then deconvolved and annotated using an open source MS-DIAL package^26^ (v4.70, RIKEN, Japan). A complete list of the software parameters can be found in Supplementary Information (Table S1). Further adjustments were performed on the raw data matrix by in-house scripts using pooled QC and diluted QC data. Briefly, intensity drift along the analytical sequence was corrected using LOESS regression^27,28^ for each feature via pooled QC (Gaussian kernel, initial span 0.75). Next, only reliable features were kept with a 2 × (dQC/pQC) ratio of 0.65 to 1.20 and relative standard deviation ratio limit of 0.2. Probabilistic Quotient Normalization (PQN) based on median QC values was applied to guarantee the comparability of the samples^29^. Annotation of steroid features was conducted according to guidelines of Metabolomics Standards Initiative^30^ using open-source retention time database DynaStI^25^. The sum of the peak areas of all adducts of each annotated steroid was chosen as the resulting relative concentration measure for the processed data matrix^31^.

### Statistical analysis

Multivariate analysis was conducted using SIMCA 16 (Umetrics Sartorius Stedim, Umeå, Sweden). Data were further preprocessed by mean centering and unit variance scaling. Model validation was carried out using leave-one-out cross-validation. Orthogonal partial least squares (OPLS) prediction performance was assessed using the correct classification rate and the discriminant Q^2^ (DQ^2^) index^32^, an adaptation of the standard Q^2^ value to discriminant analysis. The Ward method based on Euclidean distances was used for hierarchical cluster analysis (HCA).

## Results

### Analytical process

To study alterations in a systemic extended steroid profile during prostate cancer progression blood plasma was collected from four different groups of patients: healthy controls, diagnosed with benign hyperplasia, intraepithelial neoplasia and prostate cancer. Blood samples were taken prior to any medical treatment related to PCa to avoid major bias originating from a pharmacological action of the drugs. The total steroid content of the plasma was extracted by solid phase extraction (SPE) to decrease unwanted interferences and reduce matrix effects.

**Figure 3:**
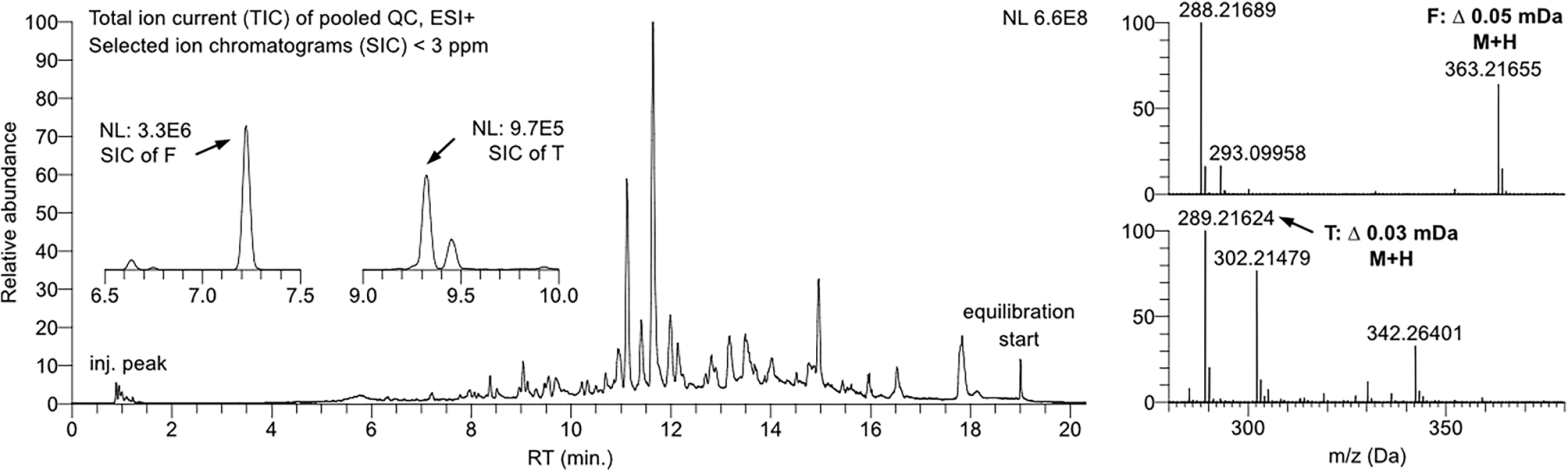
Total ion current (TIC) of a pooled QC sample with selected ion chromatograms and corresponding mass spectra of M + H adducts of cortisol (F) and testosterone (T). The observed mass accuracy was 0.05 mDa (0.14 ppm) for F and 0.03 mDa (0.1 ppm) for T.

**Figure 4:**
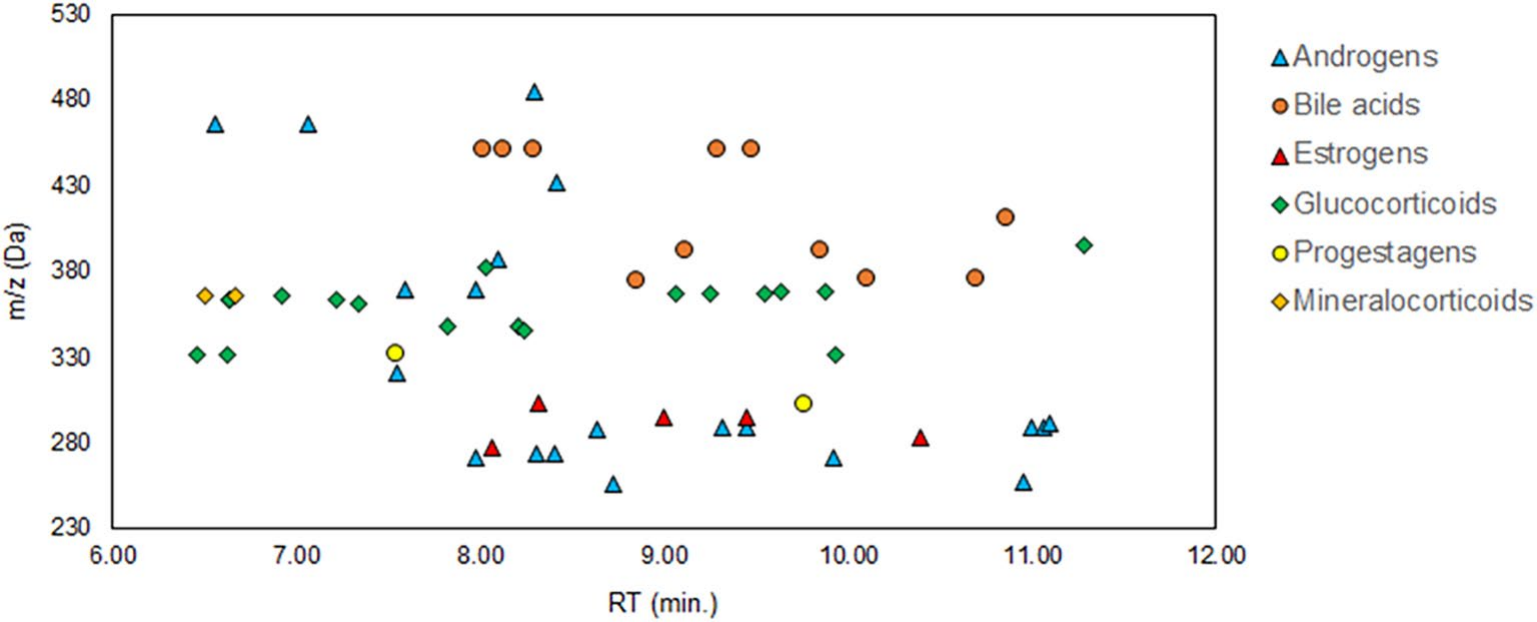
Map of steroid metabolites annotated in the region of 6–12 min/230–530 Da.

Linear gradient of the DynaSTi method, initially designed for untargeted screening, provided a separation of steroids in a well-defined elution region between 6 and 12 min, with the exception of hydroxy-cholesterols, which eluted closer to the end of the gradient. Absence of signals in the beginning of the chromatogram demonstrated successful removal of salts, hydrophilic peptides and other metabolites with lower retention. Resolution of important steroids, such as testosterone, from their isomers was achieved. Internal calibration in each acquired scan secured a sufficiently low mass error (average values of 0.3 mDa/0.9 ppm) to obtain selected ion chromatograms (SICs) for a stringent RT matching (Fig. 3). Tradeoff between reliable compound annotation and computational bias due to insufficient compound occurrence was resolved by setting of the feature acceptance only by 100% occurrence in any study group with inclusion of pooled QC samples. Hence, 19 steroids could be unequivocally annotated at Level 1 with retention time tolerance of + /- 2%, whereas 15 steroids were annotated at Level 2 (incl. Level 2 +) either as a summary annotation due to a close proximity to the database RTs for both candidates or RT for a candidate being a prediction from SRR model. The remaining 26 entries annotated as steroids isomers at Level 3, as the AM from the database produced a consistent chemical entity in the corresponding elution region across the dataset. Therefore, the probability of such a detected compound to be a steroid was considered sufficient. In total, all accepted annotations belonged to androgens (20), bile acids (11), estrogens (5), progestogens (2), glucocorticoids (18) and mineralocorticoids (2), together with cholesterol derivatives (2) (Fig. 4). A comprehensive list of the identified or annotated steroids with the corresponding figures of merit can be consulted in Table S2.

### Statistical analysis

**Figure 5:**
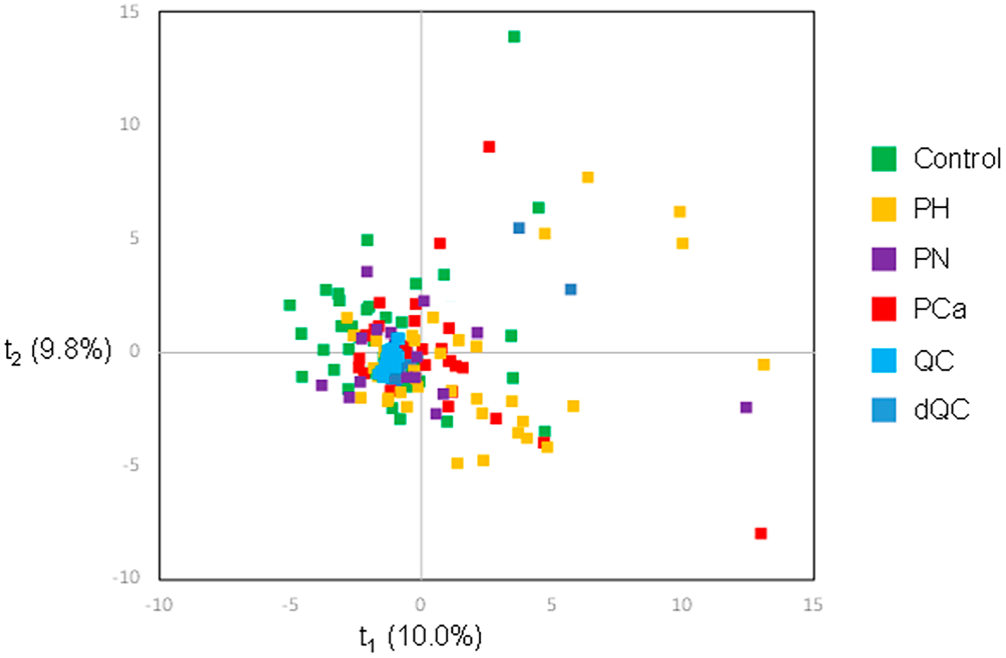
PCA score plot showing the major trends of variability among pooled QC and the four study group samples.

An initial exploratory evaluation of the processed data was performed using principal component analysis (PCA). The score plot revealed an overlap of four studied groups on the first principal plane summarizing about 20% of the total variance (PC1 10.0%, PC2 9.8%). A trend of separation along the second principal component could, however, be observed mostly involving PH and PCa groups vs healthy controls (Fig. 5).

**Figure 6:**
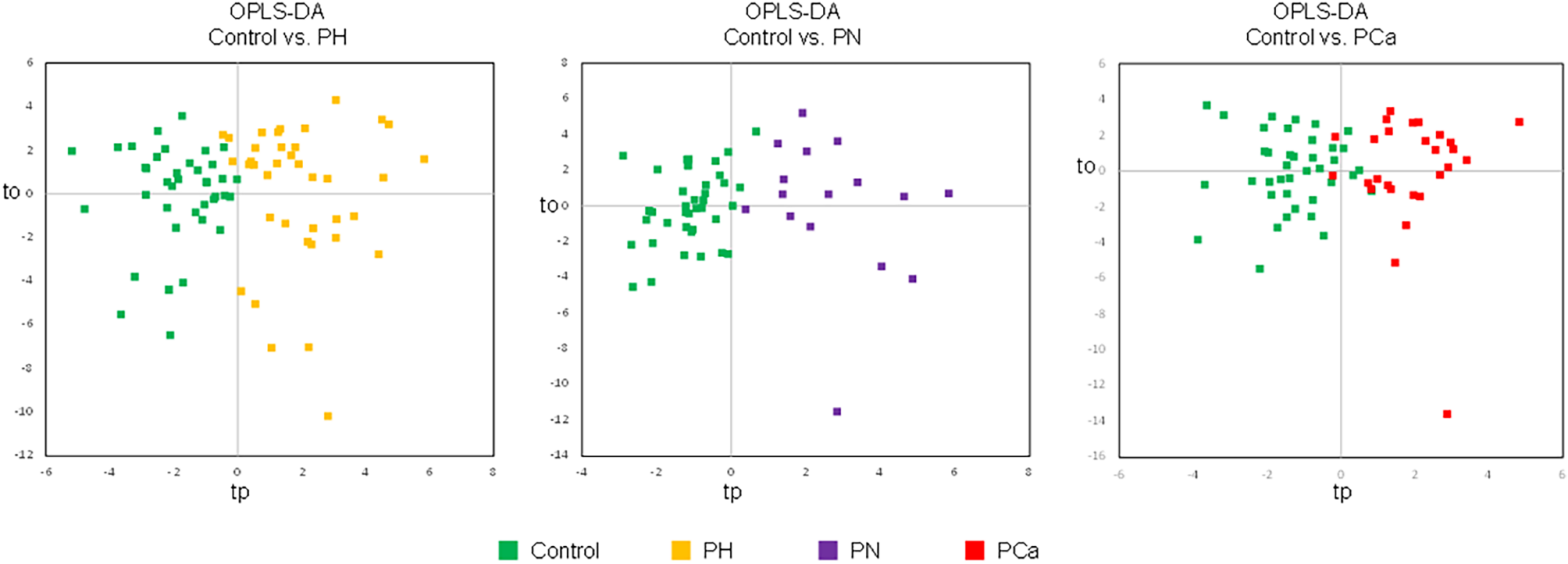
Binary OPLS-DA score plots resulted in a well-defined separation between healthy controls and patients at each stage of the disease.

The loadings highlighted minor contributions of androgens to the observed PCA separation being slightly increased in the groups with pathological status. Hence revealed patterns included dehydroepiandrosterone sulfate (DHEAS), testosterone (T) and dehydroandrosterone. Another remarkable group of steroid compounds consisted of corticosterone and its downstream derivatives such as 5a/5b-dihydrocorticosterone, one of its unknown isomers, 11-deoxycorticosterone and 19-oxo-deoxycorticosterone. Loading plots can be consulted in Supplementary Materials (Fig. S1). Binary comparisons were next conducted using supervised analysis to put emphasis on differences between experimental groups. Orthogonal partial least squares discriminant analysis (OPLS-DA) resulted in a well-defined separation between healthy controls and patients manifesting each severity stage of the disease. The models demonstrated moderate prediction ability (0.49 < DQ^2^ < 0.52) and a satisfactory accuracy of 83.6% (Fig. 6). Remarkably, shared-and-unique structures (SUS) plots derived from the OPLS-DA results highlighted similar relationships between healthy controls and all subtypes of patients. All the observations showed a similar trend, being located on the main diagonal of the plots (Fig. S2). Therefore, no pronounced differences associated with the progression of the disease could be revealed via the systemic extended steroid profile. On the other hand, the pathological status related to the prostate has a definite reflection in circulating steroid pattern.

**Figure 7:**
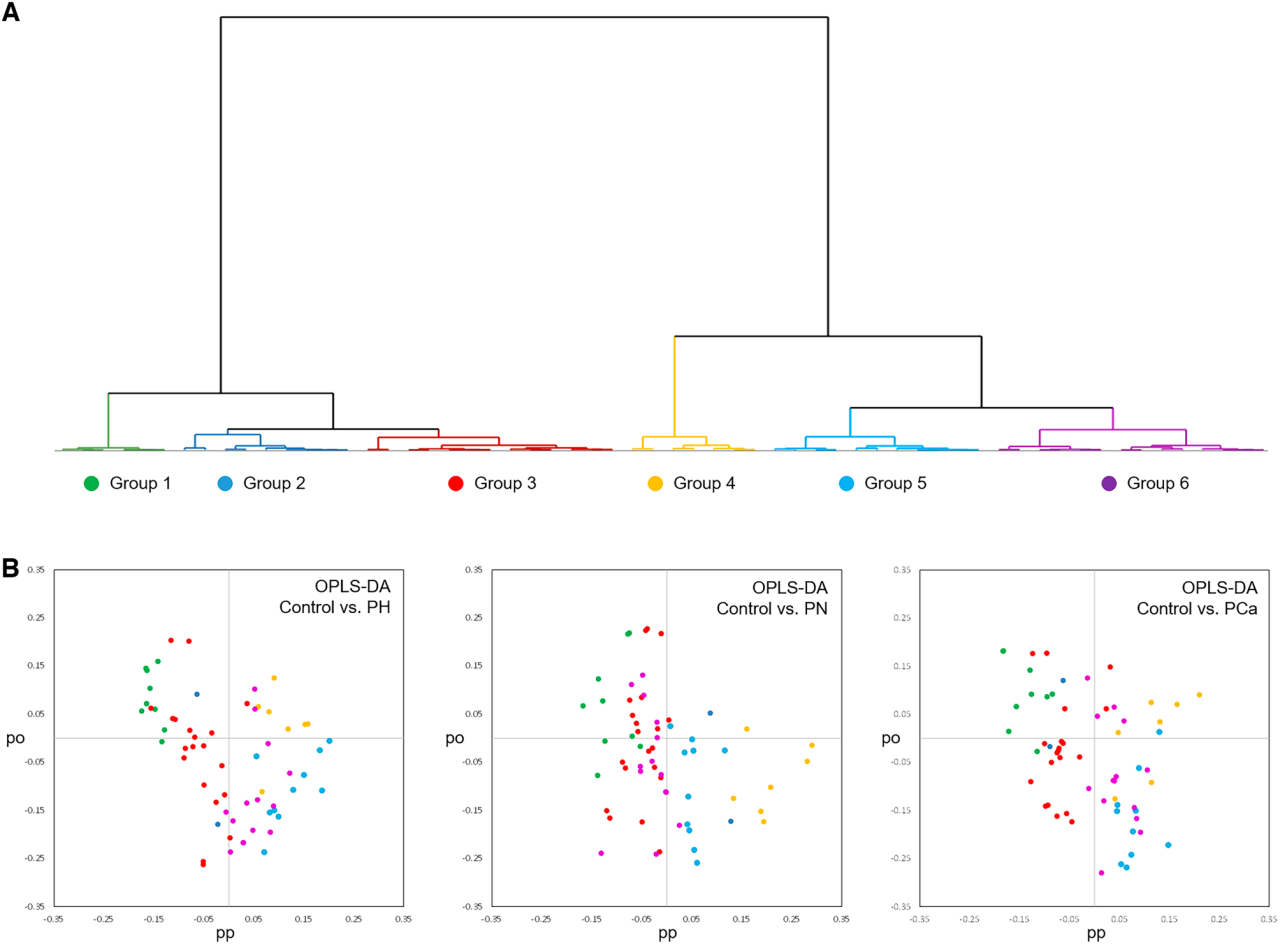
(**A**) HCA dendrogram, (**B**) Binary OPLS-DA loading plots colored according to HCA clusters.

To further explore the OPLS-DA results, HCA was performed on the concatenated matrix of predictive loadings from the three models, which revealed six distinctive groups of steroid metabolites (Fig. 7A). Next, clusters were displayed on the separate OPLS-DA loading plots to reveal subgroups of steroid metabolites underlying differences between healthy controls and different stages of PCa development (Fig. 7B). Two groups of the annotated steroids similarly defined the separation observed in OPLS-DA for all pathological conditions *vs* controls: Group 4 (T, DHEAS, isomer of deoxycorticosterone acetate, glycochenodeoxycholate and two unknown isomers (T-17b-glucuronide iso. A; 17b-OH-5b-estran-3-one is. A) and Group 5 (corticosterone (CORT), metabolites of CORT, and unknown isomers (T, T is. A, tetrahydro-11-desoxycortisol + is. A and B, dihydrocorticosterone is B). This result was in line with the information obtained from the unsupervised approach. Comprehensive outline of the HCA can be consulted in Supplementary Material, Table S3.

## Discussion

Our findings suggest the involvement of the established androgen-dependent pathway of the PCa development, resulting from activation of the hypothalamic–pituitary–adrenal (HPA) axis to increase the production of androgens required for tumor growth^33,34^. This pathway is represented by Group 4 from cluster-colored loading plots representations. This group contains T, DHEAS and a presumable isomer of T-glucuronide among others. Unfortunately, no feasible information could be collected on DHT, which is subsequently produced from DHEA and T and is an endpoint of the HPA pathway in PCa. DHT is known as a major trophic hormone for prostate tissue and a contributor to tumor growth^35,36^. Significant systemic increase of this steroid of extremely low abundance accompanied by dysregulated T and/or DHEAS would strongly suggest a heightened activity of this pathway thus increasing the diagnostic value of the extended steroid profile. Signals corresponding to this androgen were observable in the pooled QC data at the expected retention time, albeit at a very low normalized level (NL) of ca. 900 arbitrary units; however, no acceptable SICs with a well-defined peak shape could be produced with exact masses of the relevant adduct ions. It was hence not possible to distinguish DHT from a complex biological matrix. The same is applicable to a DHT glucuronide, which could be a possible proxy for this hormone due to DHT interconversion after entering the circulation^37^. In addition, Group 4 contained glycochendeoxycholate, which, along with its sulfated form, was suggested as a biomarker in previous metabolomics studies related to PCa^38,39^. Statistically significant changes for other bile acids, such as glycocholic, taurocholic and chenodeoxycholic, were reported in a urine study by Liang et al.^40^. It was also demonstrated in animals that androgens serve as PCa promotors even in near-physiological concentrations^41^. Consequently, the whole pathway should be evaluated simultaneously to elucidate the pattern of changes in the levels of relevant steroids. Secondly, individual variation in concentrations of the steroid hormones may be hardly distinguishable from overall biological variation in the cohort, which represents a strong confounding factor in this study. Hence, it is of great interest to be able to monitor also longitudinal individual changes in the systemic steroid content as recently demonstrated in a clinical application to women steroid profiles in urine and blood^42^. The resulting combination of both inter- and intra-individual observations is expected to produce a better outlook on the contribution of steroids to PCa development.

Another remarkable metabolite in the Group 4, highlighted by the unsupervised approach, was a 2-hydroxyestradiol-3-methylether (2OH3MeOE2), a compound strongly similar to 4-methoxyestradiol (4MeOE2). The concentration of the latter was reported to be eightfold higher in cancer tissues compared to a physiological norm^43^. Lacking biological activity itself, 4MeOE2 is a downstream derivative of 4-hydroxyestradiol (4OHE2). The latter is produced from E2 via CYP1B1 (also known to be increased in various types of cancer). Quinone/semiquinone forms of 4OHE2 are capable of redox cycling and generation of reactive oxygen species, leading to DNA damage and ultimately to the development of cancer^43^. A necessity of co-participation of estrogen metabolites for the development of PCa via a similar mechanism was also mentioned in Ref.^44^. Circulating E2 levels were also reported as a better predictor of high-grade PCa compared to those of T^45^. Although 2OH3MeOE2 currently holds a “predicted” status in the DynaSTi database, Lee et al. quantified this compound in a urine of females with breast cancer and prolactinoma. However, there were no evidence of it being a distinctive biomarker for these pathologies^46,47^. To our best knowledge, no data were reported on the occurrence of 2OH3MeOE2 in men.

Role of Group 5, consisting primarily of glucocorticoids (GCs), such as CORT and its downstream metabolites remains elusive. Several sources point out GCs receptor in PCa cells being able to substitute androgen receptor for the purpose of growth and proliferation^48,49^. Else, glucocorticoids can promote tumor growth via mutant androgen receptor^50^. In particular, an adaptation of GR to provide bypass for tumor growth is known for patients progressing to castration resistant PCa (CRPCa) stage as a consequence of androgen deprivation therapies, surgical or chemical castration^49–51^. Otherwise, corticosteroids, including endogenous ones, act against tumor growth and exert apoptotic and anti-inflammatory action^52^. In this study, an overall decrease of circulating corticosterone and its metabolites was observed in the steroid signatures of pathological conditions vs healthy controls. As the participants were untreated, we can hypothesize, that current observations are an indicator of disease development by the established AR-mediated pathways rather than a sign of innate GR receptor involvement. Otherwise, the observed levels of CORT and its metabolites were expected to be elevated in pathological condition, reflecting the stimulation of adrenals to provide corticosteroids needed for an active tumor growth. Androsterone, contributing to a CRPCa via one of the alternative pathways of AR signaling^53^, was also detected in lower abundance for all pathological states compared to controls, further supporting this hypothesis.

In addition, CORT was recently mentioned in a communication by Mahmud et al. as a good candidate for a prognostic PCa marker in the systemic circulation. A marked elevation was observed in urine samples of a small cohort containing patients with prostatitis (11), PH (23) and PCa (26) *vs* healthy controls (16)^54^. On the other hand, glucocorticoids are highly involved in regulation not only on a tissue level (e.g. tumor microenvironment), but provide a sound contribution to the state of the whole organism through the effects on general immunity and metabolism^55^. Therefore, a more complex longitudinal study including controls of different age/health status (ex. those manifesting hypertension, but not any of the PCa stages) would be beneficial to obtain a higher level of detail regarding GCs patterns in PCa progression.

## Conclusions and outlook

This study demonstrated that the subtle alterations in the systemic steroid profile indeed reflect the pathological status of the patients towards PCa. However, no specific steroid or group of steroids could be suggested as a definitive marker of the progression of the disease. A dysregulation of the steroid pathway belonging to the HPA axis was clearly observed, which is in line with current knowledge. Moreover, a yet unexplained participation of CORT and its metabolites could be detected. Based on these findings, we hypothesize that the search for a defined biomarker may be rather misleading for the global extended steroid profile, as (a) steroids are known to contribute to the development of PCa in near-physiological concentrations and (b) inter-individual variability can mask alterations of the circulating steroid pattern. Instead, an addition of longitudinal monitoring would be favorable to reveal a baseline pattern of relative steroid concentrations for each participant. These observations may allow for an adjustment of the resulting global steroidomics to reduce bias of an intergroup comparison and generate a reliable biological hypothesis. On top of that, contribution of estrogens in men appears to be of a particular interest in case of PCa, as the subtle separation between study groups observed in PCA was in part driven by 2OH3MeOE2, a possible downstream metabolite of 2OHE2.

From a technical point of view, current results imply a strong demand to extend the dynamic range of untargeted HRMS-based steroidomics to the low abundance species such as estrogens and DHT. Thus, obtained data are supposed to provide a better outline of the relevant biochemical pathways. Prospective methods may include setups with dedicated means of increasing sensitivity up to several orders of magnitude. Derivatization would be undesirable for clinically-relevant higher throughput scenarios, as it is time consuming and can introduce analytical bias. Miniaturization of the chromatography^56^ in combination with mobile phase modifiers to enhance ionization of underivatized steroids, such as NH_4_F^57^ appear a better fit for purpose. On top of that, increased ion currents in the microflow regime may be beneficial for structural studies employing both traditional (e.g. collisionally-induced dissociation) and alternative fragmentation modes^58^ to reveal an identity of the unknown steroid isomers observed in RT/AM measurements in this study.

### Supplementary Information

Supplementary Figures.

Supplementary Tables.

## Data Availability

Data are available on request from the corresponding author.
