# Supplementary material for: Comprehensive plasma steroidomics reveals subtle alterations of systemic steroid profile in patients at different stages of prostate cancer disease
Source: Sci Rep. 2024 Jan 18;14:1577. doi: 10.1038/s41598-024-51859-1 (PMC10796437; doi:10.1038/s41598-024-51859-1)
Supplement: Supplementary file 1 — Supplementary Figures. [file 41598_2024_51859_MOESM1_ESM.docx]

**Supplementary Information**

**Comprehensive plasma steroidomics reveals subtle alterations of systemic steroid profile in patients at the different stages of prostate cancer disease**

Sergey Girel (1)^†^, Pavel A. Markin (2)^†^, Elena Tobolkina (1), Julien Boccard (1), Natalia E. Moskaleva (2), Serge Rudaz (1)* and Svetlana A. Appolonova (3,4)

1. Institute of Pharmaceutical Sciences of Western Switzerland, University of Geneva, 1206 Geneva, Switzerland
2. World-Class Research Center Digital Biodesign and Personalized Healthcare, I.M. Sechenov First Moscow State Medical University, 119435 Moscow, Russia
3. Laboratory of Pharmacokinetics and Metabolomic Analysis, Institute of Translational Medicine and Biotechnology, I.M. Sechenov First Moscow Medical University, Moscow, Russia
4. I.M. Sechenov First Moscow State Medical University, 119435 Moscow, Russia.

† Equal contributors

*Correspondence should be addressed to the following author(s):

Pr. Serge Rudaz

Institute of Pharmaceutical Sciences of Western Switzerland,

University of Geneva,

Geneva 4, Switzerland

Serge.rudaz@hcuge.ch

**Fig. S1.** Loading plots with remarkable groups of compounds consisting of corticosterone, its downstream derivatives and HPA-related steroids (marked with red circle).

**Fig. S2.** Shared-and-unique structures (SUS) plots derived from the OPLS-DA results demonstrated similar relationships between healthy controls and all subtypes of patients
